# The dual effect of digital communication reinforcement drivers on purchase intention in the social commerce environment

**DOI:** 10.1057/s41599-023-01539-8

**Published:** 2023-02-06

**Authors:** Kareem M. Selem, Muhammad Haroon Shoukat, Syed Asim Shah, Marianny Jessica de Brito Silva

**Affiliations:** 1 Hotel Management Department, Faculty of Tourism and Hotels, Suez Canal University, Ismailia, 41522 Egypt; 2 Department of Management Sciences, COMSATS University Islamabad, Attock Campus, Attock, Punjab 43600 Pakistan; 3 Federal University of Pernambuco, Marielle Franco, Caruaru, Pernambuco Brazil

**Keywords:** Information systems and information technology, Science, technology and society, Business and management

## Abstract

The paper draws on the theory of planned behavior (TPB) to investigate the dual effect of digital communication reinforcement drivers: positive (i.e., interactivity, argument quality, hedonic motivation, and perceived enjoyment online) and negative (i.e., intrusive concerns and privacy concerns) on purchase intention. This paper also examines the mediation effect of perceived usefulness and the moderation effect of habit. Using a time-lag approach, 490 responses were collected from Pakistan’s social media users and then analyzed using SmartPLS v.3.2.8. Findings showed that interactivity, argument quality, and privacy concerns significantly affected purchase intention. Furthermore, perceived usefulness was partially mediated, and habit was discovered to be a significant moderator in liking perceived usefulness with enjoyment online and purchase intention. This paper advances TPB understanding and develops an integrated model for businesses to better understand customer physiology on social commerce platforms through effective contributions in theory and practice.

## Introduction

The COVID-19 pandemic has caused a broad and long-term problem in the retail business (Akram et al., 2021). Businesses must build the necessary resilience to sustain themselves in this new situation (Eger et al., 2021). The pandemic highlighted the importance of information technology and digital transformation, accelerating the purchase process through Instagram, Facebook, Amazon, and Pinterest (Van Veldhoven & Vanthienen, 2022). Previously, consumers shopped at shopping malls and stores, but after COVID-19, they limited their visits to stores and preferred social commerce platforms (Pillai et al., 2022).

Social commerce is a suitable setting for this study since it aims to examine consumer behavior from a contextual viewpoint, which has gotten little empirical examination (Wang et al., 2022). Retailers and consumers contribute to the growth of online shopping services (Kunst & Vatrapu, 2019). Besides, eMarketer discovered that social commerce grew by 28% in 2017 and 2.9% in 2018, and is expected to grow by 20.7% in 2019 to $3.535 trillion.

Predatory sales would have surpassed $5 trillion by the end of 2021, but growth rates would have fallen below the 20% mark beginning in 2020 (Huang et al., 2021). The data on social commerce highlights a practical gap in the social commerce context. It is a highly dynamic environment, with users’ purchasing habits changing most of the time (Akram et al., 2021). As a result, we will concentrate on the social commerce environment and investigate consumer responses to online advertising (Kunst & Vatrapu, 2019).

To cope with the changing environment, advertisers are constantly developing new methods for quickly understanding emerging trends and designing ads to elicit positive consumer responses (Zheng et al., 2019). Hence, this provides a new concept for advertisers and merchants to interact actively with consumers and track their behavior on these platforms (Wang et al., 2019). They could not previously monitor consumer behavior through traditional advertising methods (Wang & Kim, 2017). In TV advertising, advertisers could not monitor viewer behavior if they had intrusive concerns with commercials. However, in digital media, customers can reject ads if they are annoying or invasive, and advertisers can notice this directly (Li et al., 2020).

Social commerce combines online shopping and social networking, enabling native product and service purchases on these platforms (Wang et al., 2022). In addition to allowing purchases, social commerce enables the organization to communicate with the target market, establishing an exchange of information that generates interaction between them (Van Veldhoven & Vanthienen, 2022). Online networking allows for two-way communication between advertisers and customers, allowing for more rapid and richer direct consumer interactions (Shareef et al., 2018). Through better communication, these platforms may establish direct ties between buyers and sellers (Jacobson et al., 2020).

Communication is crucial to social commerce because it influences how consumers choose which products to buy (Kunst & Vatrapu, 2019). Consequently, social media is actively utilized to market items (Wang & Yu, 2017). Companies devote greater time and resources to promoting and advertising goods and services on social media platforms to enhance client purchase intention (Alalwan, 2018). Numerous studies demonstrate that social media technologies enable both online retailers and common people to engage in social commerce (Chen et al., 2017; Sarkar et al., 2020).

Some studies were conducted on social commerce advertisements through different social media platforms to evaluate purchase intention (Lee & Hong, 2016; Wang et al., 2022). However, scholars argue that the reinforcement drivers behind the ads are directly linked to the customer’s purchase decision process (Kunst & Vatrapu, 2019). These reinforcement factors are critical antecedents of purchase intention and have importance in social commerce advertisements (Goraya et al., 2021). The literature focuses primarily on positive reinforcement drivers of consumer purchase intention.For instance, Akram et al. (2021) considered hedonic and utilitarian motivation, perceived enjoyment online, and interactivity as positive predictors of purchase intention (Holdack et al., 2020).

In contrast, intrusive and privacy concerns negatively predict purchase intention (Jacobson et al., 2020). Perceived usefulness is the main element determining purchase intention, according to recent research (e.g., Alalwan et al., 2017; He & Shao, 2018). As a result, we looked at perceived usefulness as a potential mediator between reinforcement drivers and purchase intention. Ads offered on these platforms are a new medium of communication that requires more research to recognize that individuals can economically and instructively communicate with one another by giving reviews, commenting on, and sharing the ads, thereby establishing an online consumption and communication habit (Chen et al., 2017). Hence, the research questions listed below must be answered:

RQ1: How can positive and negative ad reinforcement be deployed to increase consumer purchase intention?

RQ2: How can perceived usefulness mediate the ad’s drivers and purchase intention linkage?

RQ3: How can consumer habit moderate the perceived usefulness-purchase intention linkage?

The objectives of this paper are threefold: first, to investigate the effect of ad drivers on purchase intention; second, to recognize positive reinforcements, i.e., interactivity, perceived usefulness, hedonic motivation, and argument quality, and negative reinforcement (intrusiveness and privacy concerns) (Alalwan et al., 2018; Feng & Xie, 2019); third, to examine the mediating role of perceived usefulness in the association between ad drivers and purchase intention (Luo et al., 2020); and fourth, to examine the moderating effect of habit (Sharifi Fard et al., 2019; Gardner et al., 2020).

From a theory of planned behavior (TPB), we created a fictitious model (Ajzen, 1991; Venkatesh et al., 2003). It is important to note that this study applied social media’s digital reinforcement drivers as contextual elements of TPB theory. In addition to examining the mediating role of perceived usefulness and the moderating role of habit among consumers’ purchase intentions, this offers deep insights to social media advertisers into how positive reinforcement and negative enforcement drivers are regarded as contextual factors in the TPB model.

We present a variety of theoretical advancements and their applications to digital reinforcement with social commerce advertising intentions. First, this study adds to our prior understanding of contextual factors influencing consumer purchase intention as influenced by social media reinforcement, evaluating both positive and negative reinforcement is evaluated. Although earlier research in the social commerce context has mainly addressed these concepts in isolation (Wang et al., 2019), research that integrates these concepts into a coherent nomological network is unknown, as this paper investigates. Second, there is a dearth of empirical evidence about modifications in consumer behavior.

By discussing the TPB theory (Alzahrani et al., 2017), we used extensive literature to define these notions, operationalize them, and empirically evaluate variances in individual behavior. Third, our research contributes to the body of research on TPB, which has been used to explain and forecast behavioral intention and individual behavior in the past (Cooke & French, 2008). Although the majority of recent empirical research has concentrated on the factors that influence purchase intention, little is known about how positive and negative reinforcement differ. By filling these three gaps, we intend to broaden the knowledge of TPB theory.

Finally, this paper helps social commerce advertisers efficiently design ads to forecast consumer purchase intentions. For example, when creating ads, advertisers should analyze the fit between their specific social media ads and consumer response to advertisements regarding intrusive concerns, perceived enjoyment online, hedonic motivation, and argument quality.

## Literature review and hypothesis development

The degree to which a person thinks that utilizing a certain system, example, or service might boost their effectiveness in a particular task is referred to as perceived usefulness (Davis, 1989). In the context of internet technology, perceived usefulness plays a significant role in the behavioral intention of consumers when they purchase online (Davis, 1989). Sohn (2017) revealed that perceived usefulness plays a pivotal role in forming positive intentions toward social media platform usage. Moreover, purchasing online would increase customers’ efficiency, strongly impacting their entire purchase process. However, perceived usefulness affected purchase intention; for instance, Nysveen et al. (2005) found this relationship insignificant, while Sohn (2017) found a significant relationship between them. We classify it as a contextual component of the TPB theory based on arguments and seek to examine it as a mediator between positive and negative reinforcement drivers and purchase intention in the social commerce setting.

One basic assumption is that perceived usefulness and purchase intention are interlinked (Baker-Eveleth & Stone, 2020). The theory of reasoned action (TRA) provides additional support for this supposition by explaining how the behavioral goal defines the attitude and personal internal beliefs of perceived usefulness (Alzahrani et al., 2017). For instance, websites selling event tickets must consider how customers assess the visual quality, perceived usefulness for information search, and purchase intention while evaluating online shopping platforms supplying clothing (Natarajan et al., 2017). According to the literature, perceived usefulness and purchase intention are related (Davis, 1989; Holdack et al., 2020). Hence, we made the following proposal:

H1. Perceived usefulness is positively related to purchase intention.

One of the most important parts of social commerce is customer interaction with social media platforms, which is called interactivity (Alalwan et al., 2018). Interactivity significantly changes transmission approaches and how information can be perceived as valuable among online communities and transmitted to consumers regarding purchase intention (Sreejesh et al., 2020). This interactive approach enables an immediate response to physiological reinforcement changes in behavior (Li et al., 2020). For instance, interactive information through the advertising of Apple phones will enable customers to evaluate the brands against other brands and share the usefulness of information with their online communities (Alalwan, 2018). This means that interactivity leads to purchase intention and builds connection and transmission processes among individuals and advertisers (Alalwan et al., 2017).

Alzahrani et al. (2017) assert that the logic of the TPB theory demonstrates that a particular individual behavior is strongly and favorably connected to its corresponding behavior. However, there has not been much discussion in the existing research on whether this specific behavior may explain or anticipate how helpful people interpret information (Shareef et al., 2018). Customers are likely to interpret information from official brand pages or websites in the context of social commerce (Van Veldhoven & Vanthienen, 2022). Interactivity with these websites or the brand pages allows consumers to perceive the usefulness of the information and share the information among peers regarding the brand or product (Alalwan et al., 2018). We investigate whether social commerce consumer interactivity directly or indirectly reinforces the consumer’s purchase intention. Therefore, we proposed the following hypotheses:

H2a. Interactivity positively affects purchase intention.

H2b. Perceived usefulness mediates interactivity and purchase intention relationships.

Hedonic motivation is described as intrinsic and extrinsic (Sharifi Fard et al., 2019). In social media ads, consumers love the visual depiction/symbolism of different products, perceive their significant usefulness, and are outwardly engaged, bringing positive feelings and increasing purchase intention. Hedonic motivation was taken into account by Venkatesh et al. (2012) as a component of a unified theory of acceptance and use of technology (UTAUT2). However, our study considers hedonic motivation a contextual factor of TPB theory because intrinsic motivation can trigger individual behavioral intention (Akram et al., 2021). Moreover, hedonic motivation deliberates the rigorous cognitive processing and further perceives the technology’s usefulness (Tyrväinen et al., 2020; Venkatesh et al., 2003).

Prior scholars rarely described hedonic motivation in conjunction with perceived usefulness in predicting consumer attitude and behavior, mediating hedonic motivation and purchasing intention (Akram et al., 2021). Tyrväinen et al. (2020), for example, investigated hedonic motivation in relation to customer experience and behavioral loyalty and discovered a significant relationship between them. However, how this motivation drives the consumer’s use of information is still blurred in the literature (Holdack et al., 2020). However, while the link between hedonic motivation and perceived usefulness in online shopping is well-established (Sharifi Fard et al., 2019), there is less understanding in the literature on the linkage of hedonic motivation with perceived usefulness in a social commerce context (Tyrväinen et al., 2020). Hence, the following hypotheses are brought forth in this study to bridge this knowledge gap:

H3a. Hedonic motivation positively affects purchase intention.

H3b. Perceived usefulness mediates the hedonic motivation and purchase intention association.

Argument quality is also a positive reinforcement factor and refers to “the persuasive strength of arguments embedded in an informational message” (Kim et al., 2016). As identified by dual-action models, user decision-making relies upon perceiving information (Kunst & Vatrapu, 2019). As per this model, the quality of information is a significant sign that substantially impacts communication and leads to purchase intention. Customers are more attentive to information that depicts product or service details (Xu & Yao, 2015). For instance, Kim et al. (2016) found that a stronger argument quality is positively correlated with a stronger perception of the message’s usefulness. To understand purchasing intention, information standards and the quality of relevant information are therefore of extraordinary relevance during the message information perception process (Haque et al., 2018).

Prior scholars demonstrated that the perceived usefulness of technology came before argument quality (Baker-Eveleth & Stone, 2020; Holdack et al., 2020). Recently, Kim et al. (2016) explained that when the ad’s content describes the complete product features in detail and the usefulness of products, consumers can perceive a higher quality of content (Alalwan, 2018). This also highlights the argumentative quality of content offered in social media ads. However, the literature rarely discusses the mediation effect that identifies the perceived usefulness of ads and predicts consumer purchase intention. Haque et al. (2018) recently highlighted the research gap in the linkage of argument quality with purchase intention. Consequently, the following hypotheses are brought forth:

H4a. Argument quality positively affects purchase intention.

H4b. Perceived usefulness mediates the argument quality and purchase intention association.

Perceived enjoyment online is defined as a customer’s discernment that purchasing on the internet will be fun (Alzahrani et al., 2017). As pleasure is an appropriate reaction and influences perceived enjoyment, shopping online is a crucial aspect that determines purchase intention (Natarajan et al., 2017). Though, customers can have a great time looking for products on the internet (Goraya et al., 2021), importance should be given to this factor when intending to build up their ads for social commerce platforms, as perceived enjoyment online significantly affects online shopping and increases purchase intention (Smink et al., 2019).

In contrast to offline purchases, online purchases can be equally pleasurable and have a positive impact on online purchases (Holdack et al., 2020).Perceived enjoyment online differs from hedonic motivation in that hedonic motivation provides consumers with both intrinsic and extrinsic excitement (Zheng et al., 2019). In contrast, perceived enjoyment online provides an overall judgment of a specific advertisement offered on social commerce platforms (Holdack et al., 2020).

Recent studies showed that each user’s online experience should take into account perceived usefulness and satisfaction (Smink et al., 2019). For example, developers of social commerce websites and retailers looking to enter “*online shopping platforms*” would prioritize satisfaction, which has a significant and positive impact on buying apparel and making purchases on brand websites (Goraya et al., 2021). This may be accomplished by increasing consumer perceived enjoyment online (emphasizing the ability to purchase from anywhere or benefit from in-store promotions), as buyers are increasingly seeking hedonic elements (Natarajan et al., 2017). However, it is necessary to address the dearth of research on the connection between online perceived enjoyment and purchase intention in social media advertising (Alalwan, 2018). Furthermore, research on the mediating function of perceived usefulness between perceived online enjoyment and purchase intention is scarce. As a result, we put up the following hypotheses:

H5a. Perceived enjoyment positively affects purchase intention.

H5b. Perceived usefulness mediates the perceived enjoyment online-purchase intention association.

Intrusive concerns are a part of security in online business (Morimoto & Macias, 2009). When individual customers’ online promotions are customized, it creates a perception of intrusive concerns that previous studies have related to online advertising (Maduku, 2020). For example, seeming intrusive concerns have a negative influence on consumers’ perceptions of visited sites and negatively affect purchase intention (Feng & Xie, 2019). There is a scarcity of studies to evaluate customer intentions toward ads. Consequently, the current paper reveals little insight into how apparent intrusive concerns may affect customer purchase intention toward these internet-based purchase practices (Jung, 2017). Hence, the following hypotheses are proposed:

H6a. Intrusive concerns negatively affect purchase intention.

H6b. Perceived usefulness mediates the intrusive concerns-purchase intention association.

According to Burgoon et al. (1989), privacy is the power to manage and restrict who has access to you physically, socially, psychologically, and informationally. Access to personal data in an online setting is closely related to privacy concerns. Cheah et al. (2020) underlined that privacy concerns should not be ignored in online shopping since they can harm consumer trust and purchase intention. Unlike the positive responses to linked marketing messages, the personal life issue negatively impacts advertisement performance. People with a high level of privacy had a negative attitude toward direct marketing, which negatively affected purchase intention (Jung, 2017).

Furthermore, privacy concerns are always a growing issue, and they inevitably stifle the growth of online retail (Cheah et al., 2020). This has been the most challenging task for retailers: giving their customers privacy. Privacy concerns have rarely been discussed in the literature on consumer behaviors. For example, future research must examine whether consumers make ongoing purchases with confidence in their privacy protection (Bansal et al., 2015). It is unknown how customers see these privacy concerns’ usefulness in their purchase behavior. For instance, privacy issues may be resolved by protecting and upholding customers’ information rights and putting in place a privacy policy that gives them control over how their personal information is collected and used (Cheah et al., 2020).

Privacy concerns are regarded as a detriment to the new online social life (Maduku, 2020). Customers are more concerned about their privacy when liking and sharing posts and videos (Inman & Nikolova, 2017). Jung (2017) recommends that privacy concerns be discussed in conjunction with the perceived usefulness of technology. In this study, negative reinforcement worries have been investigated as a possible barrier to online business expansion, predicting consumers’ purchase intentions regardless of how they view the advantages of technology. Therefore, the importance of privacy concerns raises advertising insecurity and significantly affects consumers’ advertising intentions (Baek & Morimoto, 2012). It is evident that privacy concerns are a growing phenomenon and need to be discussed in social commerce ad contexts (Kunst & Vatrapu, 2019). Hence, we proposed the following hypotheses:

H7a. Privacy concerns negatively affect purchase intention.

H7b. Perceived usefulness mediates the privacy concerns-purchase intention relationship.

Habit is defined as “*the degree to which users can act because of their learning automatically*” (Venkatesh et al., 2012). People get a more typical social media course of action from the social media ad environment. They primarily use social media channels for daily interaction in their marketing activities (Alalwan et al., 2017; Shareef et al., 2019). Venkatesh et al. (2012) considered habit and hedonic motivation in the UTUAT2. Prior research suggested that habit should be considered with TPB theory to advance understanding of UTUAT2 (Alalwan, 2018). We believed consumer habits to be a contextual factor in TPB theory and assumed that they caused consumers’ purchase intentions.

According to Sharifi Fard et al. (2019), there is a real connection between purchase intention and social media platforms, and researchers have discovered the habit-moderating relationship between perceived usefulness and purchase intention (Khalifa & Liu, 2007). These researchers contend that consumers’ online purchasing habits may affect the consequences of these purchasing factors (Alalwan, 2018). Additionally, they showed that the relationship between perceived usefulness and purchase intention in the context of social media advertisements had received less attention (Sharifi Fard et al., 2019). The relationships between perceived usefulness and purchase intention are therefore moderated by habit. As a result, the following claim is made:

H8. Habit moderates the usefulness-purchase intention linkage.

**Fig. 1:**
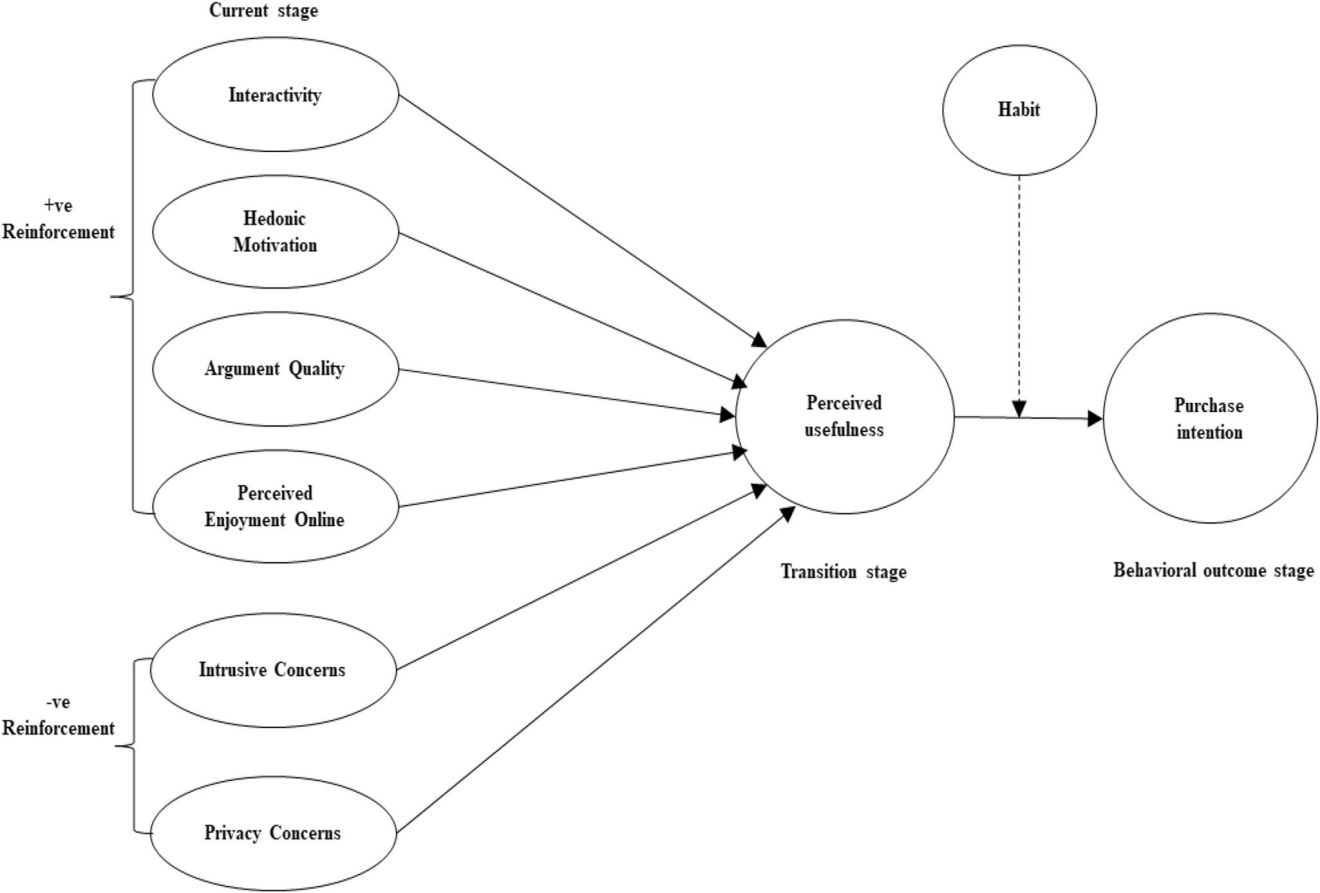
Research model between digital communication drivers and purchase intention.

In a social commerce setting, the link between digital communication drivers and purchase intention is shown in Fig. 1. This figure also examines the mediating roles of perceived usefulness and habit.

## Methods

### Sampling and data collection

The current paper’s respondents are social media users in Pakistan. The reason for selecting social media users as the target population is to describe the best ads’ behavioral response (Alalwan, 2018). Accordingly, the purposive sampling technique was applied. Respondents were approached via Google form link, WhatsApp, and Messenger. A pilot test containing 50 respondents was conducted to ensure the survey’s clarity. Customers who had previously utilized social media sites were surveyed using a Google form platform. Data were collected from October to December 2021 using a time-lag approach with three waves. This approach was used to reduce “common method bias” issues. Each data collection wave was conducted over an entire month and included three constructs in the intended survey.

The final sample size was 490 out of 700 questionnaires distributed in this survey after omitting incomplete questionnaires containing outliers. The necessary sample size was determined using G*Power v3.1.9.7. According to Faul et al. (2009), the sample size of this inquiry is sufficient for carrying out statistical procedures since the F-test with multiple linear regressions was validated. On the other hand, a reverse translation approach to the questionnaire was also conducted from English to Punjabi to suit the official dialect of the target respondents (social media users) in Pakistan, as suggested by Brislin (1980). All items were gauged on a 7-point Likert scale ranging from 1, “strongly disagree,” to 7, “strongly agree.”

### Common method bias (CMB)

Pre-validated scales with specific measurement items were employed, which were examined by several academic specialists and proofreaders (Podsakoff et al., 2012). Additionally, the Harman test was used as a reliable determinant in statistical remedies to confirm CMB issues (Podsakoff et al., 2003) by merging all measurement items into a single factor. Our findings revealed that the single-factor explanatory power (31.6%) is smaller than the total variance (50%) criterion, signaling no significant bias. Given this reasoning, CMB is not a significant worry in this work.

### Respondent profile

Out of 490 respondents, 411 (83.9%) were male. Most respondents lie between 21 and 30 years old (75.4%), while the youngest 20 (4.1%) fall under 20 years old. The majority of respondents (64.3%) had a bachelor’s degree. All respondents have enough social media platform experience, having used digital platforms 3 or 5 years ago (93.9%).

### Analysis strategy

Data were gathered and examined using SmartPLS 3.2.8. The PLS approach was employed because it could simultaneously portray the linkage between all latent constructs while accounting for measurement errors (Hair et al., 2019). Therefore, PLS was determined to match this paper’s objective and is explanatory. This is in line with Hair et al., 2017 suggestion that the measurement models be evaluated independently before the evaluation of the structural model. SmartPLS is a structural equation modeling (SEM) software that employs the PLS approach. SEM is a method for analyzing multivariate data. It is most frequently employed in social science research since it can evaluate additive and linear causal models that are theoretically justified (Black & Babin, 2019).

PLS, which primarily focuses on examining variances, is one of the many techniques in SEM. PLS is a simple modeling technique for SEM that often does not make assumptions about the details of the data distribution (Vinzi et al., 2010). As a result, it is fundamentally the greatest option for handling a variety of issues (Ringle et al., 2015). The PLS approach always considers the predictors’ assumptions as non-parametric and concentrates on variances, i.e., it is a predictability-focused tool. Finally, when the sample size obtained is either small or large, PLS is regarded as a reliable method (Hair et al., 2017).

## Results

### Measurement model

Utilizing the structural model’s model fit indicators, the validity of the proposed explanation was first determined. Fit measures for validity validation of the measuring model indicate satisfactory results: SRMR = 0.051, d_ULS = 1.202, Chi-Square = 3700.212, and NFI = 0.798). All these findings matched the suggested threshold, indicating the model’s good compatibility with the results (Hair et al., 2019). The measurement model’s convergent validity is shown in Table 1. All of the constructs satisfied Drost’s (2011) suggested threshold values of 0.70 for coefficient alpha and 0.60 for factor loading.

**Table 1:** Convergent validity.

Indicator	Loading
***Interactivity (INT)****.* Adapted from Haque et al. (2018) CR = 0.923; Alpha =0.919; AVE = 0.755	
INT1: Post-COVID-19, advertising on social commerce platforms effectively gets product feedback from customers.	0.816
INT2: I believe that post-COVID-19; advertising on social commerce platforms makes me feel as if it cares about its customers.	0.868
INT3: Advertising on social commerce platforms allows for two-way interaction between customers and businesses.	0.816
***Hedonic motivation (HM)****.* Adapted from Alalwan (2018) CR = 0.923; Alpha = 0.916; AVE = 0.772	
HM1: Post-COVID-19, I find that watching advertisements on social commerce platforms is motivating.	0.825
HM2: Post-COVID-19, I feel that advertising on social commerce platforms is enjoyable.	0.830
HM3: The advertising on social commerce platforms is entertaining post-COVID-19.	0.826
***Argument quality (AQ)****.* Adapted from Haque et al. (2018) CR = 0.849; Alpha = 0.817; AVE = 0.661	
Overall, the product information published on social commerce platforms post-COVID-19 is?	
AQ1: Informative.	0.812
AQ2: Valuable.	0.829
AQ3: Helpful.	0.813
***Perceived enjoyment online (PEO)****.* Adapted from He and Bond (2013) CR = 0.850; Alpha = 0.837; AVE = 0.657	
PEO1: Purchasing products advertised on social commerce platforms post-COVID-19 is pleasurable.	0.794
PEO2: I like advertisements on social commerce platforms post-COVID-19.	0.687
PEO3: Post-COVID-19, I believe purchasing on social commerce platforms after watching advertisements would interest me.	0.827
***Intrusiveness concerns (IC)****.* Adapted from Lin and Kim (2016) CR = 0.901; Alpha = 0.887; AVE = 0.693	
Post-COVID-19, I think advertisements on social commerce platforms to be:	
IC1: Intrusive.	0.836
IC2: Irritating.	0.865
IC3: Interfering.	0.833
***Privacy concerns (PC)****.* Adapted from Morimoto and Macias (2009) → CR = 0.903; Alpha = 0.871; AVE = 0.750	
PC1: While seeing advertisements on social commerce platforms, I am anxious that others may identify my activities.	0.801
PC2: I am concerned that other social media users may be aware of my social media networking activities.	0.762
PC3: I am afraid my friends will discover that I enjoy the advertisements shared on social commerce platforms.	0.802
***Perceived usefulness (PU)****.* Adapted from Lin and Kim (2016) CR = 0.918; Alpha = 0.901; AVE = 0.743	
PU1: I frequently find product or service-related information post-COVID-19 through advertisements on social commerce platforms.	0.795
PU2: The advertisement on social commerce sites would make my life easier before shopping post-COVID-19.	0.742
PU3: The advertisement on social commerce platforms would make it more productive post-COVID.	0.754
PU4: The advertisement on the social commerce network would enhance my shopping abilities post-COVID-19.	0.780
***Habit (HBT)****.* Adapted from Alalwan et al. (2017) CR = 0.903; Alpha = 0.884; AVE = 0.776	
HBT1: The use of social media advertising has become a habit for me.	0.775
HBT2: Post-COVID-19, I became addicted to advertising for shopping on social commerce platforms.	0.793
HBT3: Post-COVID-19, I always saw ads on social commerce platforms.	0.792
HBT4: Post-COVID-19, I have responded effectively to seeing advertisements on social commerce platforms.	0.763
***Purchase intention (PI)****.* Adapted from Alalwan et al. (2018) CR = 0.913; Alpha = 0.907; AVE = 0.736	
PI1: If COVID-19 continues, I expect to see product advertising on social commerce platforms in the future.	0.774
PI2: Post-COVID-19, I will recommend products advertised on social commerce platforms.	0.819
PI3: I need to see advertisements on social commerce platforms in the future.	0.773
PI4: Post-COVID-19, I intend to purchase products promoted by advertisements on social commerce platforms.	0.825

All constructs’ composite reliability values were above Churchill’s (1979) suggested level of 0.70. The average variance extracted (AVE), which was considerably more than 0.5, and factor loading were used to evaluate the convergent validity of all latent constructs (Black & Babin, 2019). Furthermore, the R^2^ values of perceived usefulness and purchase intention were.539 and.406, respectively, which were within the acceptable range (Hair et al., 2019).

For discriminant validity, we used the Heterotrait-Monotrait (HTMT) ratio criteria (Henseler et al., 2015). Table 2 demonstrates that all HTMT values fall below the suggested reflective measure threshold of.90. Hence, there was no threat related to discriminant validity in this study.

**Table 2:** Discriminant validity: Heterotrait-Monotrait ratio analysis (HTMT).

Constructs	AQ	HBT	HM	IC	INT	PC	PEO	PI	PU
AQ									
HBT	0.516								
HM	0.613	0.517							
IC	0.685	0.740	0.563						
INT	0.497	0.688	0.438	0.436					
PC	0.544	0.420	0.502	0.266	0.355				
PEO	0.710	0.545	0.691	0.374	0.520	0.791			
PI	0.549	0.578	0.655	0.432	0.466	0.243	0.442		
PU	0.491	0.470	0.459	0.554	0.520	0.314	0.350	0.485	

*AQ* argument quality, *HBT* habit, *HM* hedonic motivation, *IC* intrusiveness concerns, *INT* interactivity, *PC* privacy concerns, *PEO* perceived enjoyment online, *PI* purchase intention.

### Structural model

The direct relationship paths were evaluated and described. Hence, the results obtained from indirect relationship paths are presented in Table 3. We applied the mediation analysis procedure developed by Baron and Kenny (1986).

**Table 3:** Evaluation of the structural model.

*Model fit indicators*					
SRMR	0.051	d_ULS	1.202	Chi-Square	3700.212
NFI	0.798	*R*^2^ for PU	0.539	*R*^2^ for PI	0.406
*Direct effects*					
Hypotheses	Relationships	Paths	*f*^2^	*p*-values	Supported?
H1	PU → PI	0.306***		0.000	Yes
H2a	INT → PI	0.209***		0.000	Yes
H3a	HM → PI	−0.037		0.280	No
H4a	AQ → PI	0.133**		0.001	Yes
H5a	PEO → PI	0.185***		0.000	Yes
H6a	IC → PI	−0.006		0.898	No
H7a	PC → PI	0.119**		0.001	Yes
*Indirect effects*					
Hypotheses	Relationships	Paths	*t*-value	*p*-values	Supported?
H2b	INT → PU → PI	0.061***	3.896	0.000	Yes
H3b	HM → PU → PI	0.034*	2.441	0.015	Yes
H4b	AQ → PU → PI	−0.006	0.398	0.691	No
H5b	PEO → PU → PI	−0.005	0.516	0.605	No
H6b	IC → PU → PI	0.116***	5.318	0.000	Yes
H7b	PC → PU → PI	0.093***	5.090	0.000	Yes

***p < 0.001, **p < 0.01, *p < 0.05, non-*p* > 0.05.

*AQ* argument quality, *HM* hedonic motivation, *IC* intrusiveness concerns, *INT* interactivity, *PC* privacy concerns, *PEO* perceived enjoyment online, *PI* purchase intention.

### Direct effects estimation

The direct effects of the structural model are presented in Table 3. The findings show a highly significant correlation between perceived usefulness and purchase intention (*β* = 0.306, *t* = 8.041, *p* < 0.001), implying that H1 is accepted. Interactivity positivly affected with purchase intention (*β* = 0.209, *t* = 4.809, *p* < 0.001), whereas hedonic motivation negatively affected purchase intention (*β* = −0.037, *t* = 1.081, *p* > 0.05); thus, H2a is accepted while H3a is rejected.

H4a confirms that argument quality positively affected purchase intention (*β* = 0.133, *t* = 3.369, *p* < 0.01). Empirical evidence also validated H5a (*β* = 0.185, *t* = 4.992, *p* < 0.001), directly linking perceived enjoyment online with purchase intention. Hence, H4a and H5a are accepted. Regarding negative reinforcement drivers, the results showed that intrusive concerns do not directly influence purchase intention (*β* = −0.006, *t* = 0.120, *p* > 0.05). In contrast, privacy concerns were found to significantly and negatively affect purchase intention (*β* = 0.119, *t* = 3.211, *p* < 0.01). Hence, H6a is rejected, while H7a is accepted.

### Indirect paths estimation

Table 3 represents indirect hypotheses. According to the findings, perceived usefulness has a significant mediating effect between interactivity and purchase intention. (*β* = 0.061, *t* = 3.896, *p* < 0.001), and hedonic motivation and purchase intention (*β* = 0.34, *t* = 2.441, *p* < 0.05); hence H2b and H3b are accepted. H4b and H5b are rejected, related to the significance of perceived usefulness in mediating the argument quality-purchase intention linkage (*β* = −0.006, *t* = 0.398, *p* > 0.05), and the perceived enjoyment online-purchase intention linkage (*β* = −0.005, *t* = 0.516, *p* > 0.05). H6b and H7b are accepted, demonstrating the significance of perceived usefulness in mediating intrusiveness (*β* = 0.116, *t* = 5.318, *p* < 0.001) and privacy concerns (*β* = 0.093, *t* = 5.090, *p* < 0.001) affect purchasing intention. The results revealed that the mediation effect was significant, confirming H5b and H6b. Thus, the four accepted hypotheses partially mediated perceived usefulness (Carrión et al., 2017).

### Moderation analysis

**Fig. 2:**
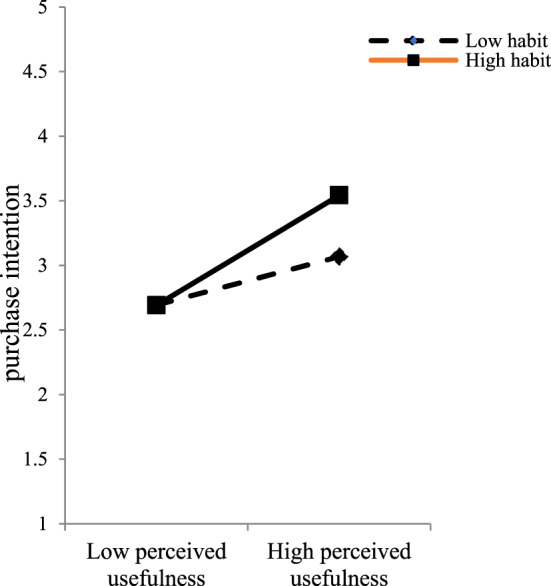
Results of the moderating role of habit when perceived usefulness has high/low levels.

We used a two-stage approach to test the moderating mechanism of habit (Becker et al., 2018). Figure 2 demonstrates a positive and significant interaction effect of habit _x perceived usefulness on purchase intention (*β* = 0.135, *t* = 4.476, *p* < 0.001). This indicates that habit strengthens the perceived usefulness-purchase intention relationship; hence, H8 is accepted.

## Discussion

### General discussion

This paper investigates how consumers’ purchase intentions in a social commerce environment are affected by both positive and negative drivers of digital communication reinforcement. These reinforcement drivers connect with consumer perceptions of usefulness, resulting in increased purchase intention. At this stage, the mediating role of perceived usefulness was investigated. Results show that perceived usefulness is the most important factor influencing the present stage. Because when consumers perceive the usefulness of positive or negative drivers, they can provide a behavioral outcome in terms of purchase intention, which may lead to the desired stage. The study’s behavioral outcomes and purchase intent were discovered.

This paper fills a research gap by addressing the question of what specific contextual variables in social media advertising affect consumer purchase intention in social commerce (Shareef et al., 2019); second, special attention should be paid to investigating the interrelationship between consumer input and behavioral outcomes of advertising drivers, with the mediating role of perceived usefulness taken into account (Yang et al., 2020); and third, how perceived usefulness affects consumer purchase intention in social commerce.

Hypotheses 2a and 3a are accepted and rejected, respectively. Interactivity had a positive, medium-sized influence on purchase intention. The findings are in line with earlier research and offer substantial empirical support (Alalwan, 2018; Sreejesh et al., 2020). Alalwan (2018) investigated how consumer interactivity with social commerce advertising is becoming a more common phenomenon and how social commerce is emerging as an appealing medium for advertising effectiveness. Their findings show that interactivity appeals are the driving reinforcement factors behind desirable behavioral responses to ads. Thus, according to our studies, consumers engage in more interactive behavior with advertisements, which leads to increased purchase intention. H3a shows that hedonic motivation is positively associated with purchase intention; however, no effect size on purchase intention was discovered, contradicting the findings of previous researchers (Sharifi Fard et al., 2019).

Hypotheses 2a and 3a are accepted and rejected, respectively. Interactivity had a positive, medium-sized influence on purchase intention. The findings are in line with earlier research and offer substantial empirical support (Alalwan, 2018; Sreejesh et al., 2020). Alalwan (2018) investigated how consumer interactivity with social commerce advertising is becoming a more common phenomenon and how social commerce is emerging as an appealing medium for advertising effectiveness. Their findings show that interactivity appeals are the driving reinforcement factors of desirable behavioral responses to ads. Thus, according to our studies, consumers engage in more interactive behavior with advertisements, which leads to increased purchase intention. H3a shows that hedonic motivation is positively associated with purchase intention; however, no effect size on purchase intention was discovered, contradicting the findings of previous researchers (Sharifi Fard et al., 2019).

Interestingly, when we incorporate perceived usefulness as a mediator, this relationship path becomes significant with a medium effect size. The findings are distinctive and contrast past research (Sharifi Fard et al., 2019). Our study used hedonic motivation as a contextual element of TPB theory that reinforces the behavioral response and influences purchase intention. For example, suppose a consumer sees an ad for an Apple phone on social media while using another variant of Apple (Mao et al., 2020).

In that case, the ad will stimulate his internal motivation to give intention to the ad and, as a result, purchase the product. In our research, we really cannot find any proof that hedonic motivation directly affects the likelihood of making a purchase. If customers are motivated and have had a favorable experience with technology, it would reinforce their intention to pay closer attention to marketing; otherwise, they would not. This would be a rejection of the hypothesis. The mediation effect of perceived usefulness on the links between hedonic motivation and purchase intention is not well understood in the literature (Yang et al., 2020).

Hypotheses 4a and 5a (H4a and H5a) are accepted, demonstrating that argument quality positively affected purchase intention with a small effect size. The findings echo those of earlier research (Xu & Yao, 2015; Haque et al., 2018). Argument quality plays a critical role in the adoption of online reviews and customer decisions, as examined by Xu and Yao (2015). Their findings show that argument quality positively affected perceived value and consumer decision-making. Handayani et al. (2020) provided more support for the adoption of mobile health applications. They discovered that people like applications when the application’s argument quality is satisfied. Argument quality is interpreted as a supportive factor of customer purchase intention in the context of social commerce.Our findings enhance our understanding of the growing concept of argument quality in an online advertising context.

Another positive reinforcement factor is perceived enjoyment online, which positively influences purchase intention. We have found a small effect on purchase intention. The findings are consistent with earlier research (Holdack et al., 2020). Results demonstrate that this positive enjoyment keeps their attention (Wang & Lee, 2020). Consequently, they are more engaged with the platform or brand. Despite previous researchers’ investigations of perceived enjoyment online in another a**s**pect of online entertainment, such as mobile applications (Goraya et al., 2021). However, this paper enhances understanding how perceived enjoyment online affects purchase intention.

Concerning negative reinforcement drives (intrusiveness and privacy concerns), hypotheses 6 and 7 (H6 and H7a) were related to the direct effects of intrusiveness and privacy concerns on purchase intention. At the same time, hypotheses 7b and 8b (H6b and H7b) were related to the indirect effect of the same drivers on purchase intention through perceived usefulness. Surprisingly, the results are unique because intrusive concerns directly influence purchase intention. However, interfering worries have a moderate effect on purchase intention when we include the indirect influence of perceived usefulness. The findings conflict with certain research and are consistent with a recent study (Cheah et al., 2020; Jung, 2017). When we add perceived usefulness as a mediator in our case, the effect becomes significant in this relationship path.

On the other hand, privacy concerns were a proposed negative purchase intention driver. This result found significant empirical evidence consistent with previous studies (Feng & Xie, 2019; Maduku, 2020). Jung (2017) examined how purchase intention in social media marketing is affected by perceived relevance and privacy concerns. Their study yielded crucial conclusions and showed that advertisers provide personalized ad messages for a person based on personal information. Hence, it may have turned into a privacy threat for consumers. Our results demonstrate that psychological reaction factors, such as intrusiveness and privacy concerns, facilitate personalized advertising involving personal information and negatively affect purchase intention.

In terms of moderation, we proposed hypothesis 8 (H8) for the consumer habit, which suggested that habit significantly moderated the perceived usefulness-purchase intention linkage. The findings demonstrate that consumer habit considerably modifies the impact of perceived usefulness on purchase intention while having a small effect size. This conclusion is consistent with earlier studies (see Alalwan, 2018b; Gardner et al., 2020). Gardner et al. (2020) revisited the habit-purchase intention relationship. They revealed that habitual behavior is recruited when a routine activity cue-behavior association that has been perceived through good performance is enlisted. This has resulted in a desire to buy particular products or brands. For example, Escobar-Rodríguez, Carvajal-Trujillo (2013) found that habit has a significant impact on a person’s likelihood and level of purchase intention when purchasing airline tickets online.

In this paper, which is unique, we propose six mediation hypotheses for positive and negative reinforcement drivers on purchase intention via perceived usefulness. The findings show that interactivity significantly affected purchase intention via perceived usefulness. This is consistent with past research (Sreejesh et al., 2020). The findings show that customers have increased purchase intentions when they interact more with advertising, at least until they appreciate the value of technology. Furthermore, hedonic motivation affects purchase intention via perceived usefulness. Surprisingly, we found no evidence of hedonic motivation having a direct effect on purchase intention. Instead, we detected a mediation effect through perceived usefulness. This means that consumer hedonic motivation does not entirely satisfy the consumer’s wants until they fully understand the value of technology, which increases their purchase intention (Akram et al., 2021).

### Theoretical implications

The collaborative nature of social commerce advertising contexts adds new facets to the interaction between digital communication drivers and purchasing behavior. The first contribution of our study is that we improve our understanding of TPB theory by investigating several behavioral reinforcement factors, particularly positive and negative, rather than a single behavioral activity. We investigated the focal effects of interactivity, argument quality, and perceived enjoyment online on purchase intention. Moreover, we investigate how these reinforcing characteristics and purchase intention are related and how perceived usefulness plays a mediating role in that relationship. Even though these variables are rarely explored in isolation, no single framework has been devised to evaluate purchase intention.

We studied missing links in the research; for example, research lacks the association between perceived enjoyment online and perceived usefulness (Holdack et al., 2020). The linkage of privacy with perceived usefulness is similarly underdeveloped (Jung, 2017). In social commerce, it has been shown that habit has a considerable moderating influence on the relationship between perceived usefulness and purchase intention.

By filling a gap in the prior literature and advancing TPB theory in the context of modern social commerce, we make a positive contribution to the field. Given the increasingly dynamic and immersive nature of customer-brand engagement in the digital age, the behavioral conclusion of this paper helps to better assess purchase intention with social commerce advertising. Our research provides in-depth insights into how positive and negative reinforcement factors can assist in the expansion of social commerce consumers’ scope of interaction with advertising beyond the prior purchase or transactional connection. It is well-established that consumers are more likely to develop a stronger bond with a brand when they see the usefulness of technology (Yang et al., 2020).

Third, we concentrated on the function of digital communication drivers in influencing purchase intention in the setting of social commerce. We chose interactivity, hedonic motivation, argument quality, and privacy concerns as the study’s contextual factors based on TPB, TRA, and UTUAT-2 theories. The topic of social commerce has grown in popularity in recent decades, particularly post-COVID-19 (Naeem, 2021). However, understanding its role as a behavioral driver is limited. As one of the initial efforts, our study helps to improve knowledge of the social commerce advertising milieu.

### Practical implications

The results of this study have practical implications for marketers that use social media as an alternative platform post-COVID-19 for advertising and marketing. First, it would seem that interaction is a crucial method for understanding the value of purchasing. Marketers must thus urge their clients to utilize their suggestions, opinions, and data to interact with social media ads more frequently. In this situation, marketers should promote communication between businesses and their clients or among customers. There may be a wide variety of material and content that is of high caliber as a consequence. When customers and the customer support staff use live text chat or chat rooms, interactions between them may improve.

Second, hedonic motivation is a critical component of social media advertisements. As a result, marketers should create their advertisements more imaginatively and innovatively, thus improving the intrinsic usefulness of such advertisements. As discussed previously, increased interaction results in hedonic motivation. Thus, interactivity with technologies can assist marketers in fulfilling the role of hedonic motivation. Hedonic motivation is increased when a combination of emotionally charged multimedia is used (such as images, music, movies, and sounds). On the other hand, it has been demonstrated that hedonic incentives significantly influence purchase intention through perceived usefulness. Therefore, marketers should give their clients the impression that these advertisements are beneficial and a good source of information for making decisions. As a result, announcements need to be more appealing and include solid, current data on consumer perception.

Third, the strength of the argument is seen as a crucial factor in social media advertising. Thus, marketers must improve the quality and volume of the information supplied. Complete and current information covering all aspects of the product, such as its characteristics, costs, special offers, supply, and availability, should be taken into account in the social media ad’s message. Any product they promote should also be centered on its value proposition. In this scenario, each commercial should attract the attention of the customer both cognitively and emotionally. Reduced costs, increased quality, customer guarantee, and product availability are all examples of promoted cognitive components. Customers’ emotions are involved in emotional elements, which are crucial for focused branding. Using a variety of mediums is crucial (video, audio, graphics, images, and text).

Lastly, purchase intention is significantly influenced psychologically by the role of the investigated digital communication reinforcement drivers, both favorably and unfavorably, and is predicted by purchase intention. Therefore, advertisers should create and customize their social media advertising to reflect the preferences and interests of their target market. Marketers should also use cookies to see customer behavior and profiles for their fans and followers. This helps marketers predict the interests and preferences of their customers.

### Limitations and future research lines

Theoretical and practical contributions are also included in this paper; however, there are many research limitations. First, this paper used a limited number of positive reinforcement drivers (argument quality, interactivity, and perceived enjoyment online) and negative reinforcement drivers (intrusiveness and privacy concerns) to evaluate consumer behavioral intentions. As the social commerce context is complex and multidimensional, other potential drivers such as trust (Sarkar et al., 2020), perceived value and perceived risk (Shaw & Sergueeva, 2019) could influence consumer behavioral intention and would be considered in future research.

Second, perceptions of a social commerce platform’s usability and convenience of use are crucial for determining a customer’s propensity to make a purchase. To date, there has been little research on this topic, and it would provide another future research direction for researchers. Third, it’s crucial to remember that the research was conducted in the setting of social commerce, which might result in generalizations; thus, this model should be replicated in other industries and sectors in future studies.

### Conclusion

To summarize, digital reinforcement drivers revealed unique purchase intentions; argument quality and perceived enjoyment online were directly significant; privacy concerns were discovered to be directly significant with purchase intention but indirectly insignificant with perceived usefulness. Few reinforcement drivers, hedonic motivation, and intrusive concerns, on the other hand, are directly irrelevant to purchase intention but indirectly relevant to perceived usefulness.This study’s findings provide empirical evidence supporting the notion that advertisers exploit these reinforcement drivers by developing tactics to analyze customer behavior to increase purchase intention. The results also demonstrate that marketers can create more informative and comfortable ads for customers to minimize intrusiveness and privacy concerns.

Given the concept of a cumulative research tradition, it is hoped that this hypothetical model will serve as a sound foundation for future studies that examine the more comprehensive conceptualization of other constructs, such as advertising skepticism (Luo et al., 2020) and attitude (McClure & Seock, 2020), to address the limitations of this modality. The results of the study may be used by managers to create a model for various customer behaviors to boost purchase intention.

## Data Availability

The datasets generated during and/or analyzed during the current study are available from the corresponding author on reasonable request.
